# Web-Based Education Program for Care Partners of People Living With Dementia (iGeriCare): Protocol for a Pilot Randomized Controlled Trial

**DOI:** 10.2196/67048

**Published:** 2025-06-04

**Authors:** Anthony J Levinson, Stephanie Ayers, Sandra Clark, Rebekah Woodburn, Maureen Markle-Reid, Brian McKenna, Doug Oliver, Alexandra Papaioannou, Henry Siu, Richard Sztramko

**Affiliations:** 1 McMaster University Hamilton, ON Canada; 2 Geras Centre for Aging Research Hamilton, ON Canada; 3 Division of e-Learning Innovation Hamilton, ON Canada; 4 Hamilton Family Health Team Hamilton, ON Canada; 5 McMaster Family Practice Hamilton, ON Canada

**Keywords:** web-based intervention, internet, eHealth, dementia, caregiver, Alzheimer disease, education and training, clinical trial, knowledge translation, randomized controlled trial

## Abstract

**Background:**

The prevalence of dementia is increasing in Canada and in many countries internationally. People living with dementia are highly dependent on family and friend care partners, who may have little knowledge of the disorder. Web-based interventions in dementia have been shown to improve care partner mental health and reduce burden, but few have been widely implemented or rigorously studied. We developed a web- and email-based dementia education platform for care partners, iGeriCare, which includes 12 asynchronous, multimedia, e-learning lessons and email-based content to reinforce the learning.

**Objective:**

The primary objective of this pilot study is to evaluate the feasibility and care partner acceptance of the intervention, including study methods. Secondary objectives will examine the effectiveness of the educational resources on family care partners’ knowledge, self-efficacy, and sense of burden.

**Methods:**

This study is a 2-arm, pilot, feasibility, randomized controlled trial. A total of 125 family or friend care partners for a person living with dementia—who are residing in Canada, aged 16+ years, and comfortable using the internet and email—will be recruited using coinvestigator networks and Facebook digital marketing advertisements. Participants will be randomly assigned to either the intervention group (receiving the dementia web- and email-based educational intervention) or the control group (receiving an alternate topic e-learning lesson and emails) and will have 8 weeks to complete baseline surveys and the assigned e-learning. After 8 weeks, participants will have 2 weeks to complete poststudy surveys. This protocol will be repeated with a second cohort of 100 care partners recruited from a paid panel service based on learnings from initial feasibility results.

**Results:**

Initial recruitment began on September 6, 2022, and concluded on October 2, 2022. A total of 125 participants were randomly assigned to the intervention (n=61) or control (n=64) group. Data collection concluded in January 2023. Preliminary feasibility results showed a substantial number of participants who did not engage with the protocol as intended. A decision was made to recruit a second cohort of participants to address these protocol deviations. Secondary recruitment began on June 12, 2023, and concluded on June 27, 2023. A total of 100 participants were randomly assigned to the intervention (n=53) or control (n=47) group. Data collection concluded in September 2023. Further results will be published in peer-reviewed journals and presented at conferences.

**Conclusions:**

This study is investigating the feasibility, acceptability, and effectiveness of a web- and email-based dementia care partner educational intervention. The results of this study will contribute to the planning of a larger randomized controlled trial in the future, as well as the evaluation of innovative, cost-effective, and efficient dementia care partner resources that can complement traditional approaches.

**Trial Registration:**

ClinicalTrials.gov NCT05114187; https://clinicaltrials.gov/study/NCT05114187

**International Registered Report Identifier (IRRID):**

DERR1-10.2196/67048

## Introduction

### Dementia Care Partners

People living with dementia rely on family care partners to provide support, most often without any formal dementia education. In Canada, at least 486,000 people identify as caring for an individual with dementia [1]; many more constitute informal family or friend care partners. By 2025, Canada will have more than 1 million care partners for people living with dementia who will provide an estimated 1.4 billion hours of care annually [1-5].

Numerous quality standards highlight the importance of education that helps care partners develop knowledge and skills to support themselves and a person with dementia in living well [1,6-8]. Providing evidence-based education to help care partners better understand dementia, its progression, treatment options, and available supports can be challenging to do in the clinical setting; time and resource constraints can impede a provider’s ability to provide education. Although several education programs currently exist, they vary in availability and often lack the flexibility to accommodate the realities of caregiving.

### Web-Based Strategies

Care partner–focused, web-based interventions have been shown to be both feasible and effective with respect to a range of outcomes. Recent systematic reviews found evidence for the positive effects of web-based interventions on self-efficacy, self-esteem, and strain of care partners of adults living with a chronic condition [9-13]. There is also evidence for the benefit of internet-based interventions on mental health for care partners of adults living with a chronic condition, particularly for the outcomes of care partner depression, stress and distress, and anxiety [14].

However, there remains a shortage of freely available, asynchronous, web-based programs that incorporate principles of multimedia learning design. Care partners of people diagnosed with dementia have identified the need for high-quality, accessible, and trusted internet resources [15]. There are several frequently unmet needs in dementia care partner education including flexible delivery methods as well as rigorous evaluation of program effectiveness [16-18]. Existing programs often rely on real-time components such as synchronous (live) webinars, which can be inconvenient for care partners and challenging to scale and spread. A recent scoping review highlighted the importance of several components of internet interventions, including evidence-based instructional design, codesigning online education with care partners and experts in dementia care, and using randomized controlled trials (RCTs) to more rigorously evaluate their effectiveness [19]. Comparable web-based educational programs in other chronic disease contexts provide valuable insights. The RHAPSODY program for young-onset dementia caregivers showed significant improvements in care partner confidence but required scheduled participation [20]. The STAR portal for dementia care partners demonstrated knowledge gains but faced challenges with user engagement and retention [21]. These experiences highlight the importance of flexible, asynchronous delivery and robust instructional design.

### iGeriCare: Web-Based Educational Program for Dementia Caregivers

iGeriCare is a free, evidence-based, web- and email-based educational program for family and friend care partners of people living with dementia. It was developed by experts in dementia, e-learning, mental health, and lived experience caregiving to help meet the needs of care partners by improving their knowledge, and to raise awareness of strategies and services to improve their self-efficacy and quality of life [22-24].

iGeriCare is unique when compared to other care partner educational resources because it is one of the few web-based dementia care partner interventions to incorporate best practices in multimedia e-learning instructional design, asynchronous lessons, and email-based microlearning while remaining open access (free of cost) and without requiring users to create an account or log-in. Key components of the instructional design include the use of the principles of multimedia learning and audio narration; personalization, including the use of a visual “coach” or embodied instructional agent; segmenting the content into manageable topics to reduce cognitive load; authentic scenarios and worked examples; interactive review questions; and learner control over navigation [22]. An iterative, participatory instructional design and development methodology, the Successive Approximation Method [23], was used, with extensive involvement and review from a range of experts in dementia care, including lived-experience dementia care partners.

The asynchronous and fully web-based elements of the intervention have proven even more valuable during the COVID-19 pandemic when many face-to-face approaches have been unavailable [24]. It is easy to implement for clinicians and is an efficient and cost-effective way to address dementia care quality standards. This study, which is the first clinical trial studying the feasibility and effectiveness of the iGeriCare platform in a clinical trial, will be an important contribution to the literature on care partner web-based interventions. These innovations are an important complement to traditional approaches to care partner education, a key component of quality dementia care.

### Research Aim

The overall aim of this study is to explore the feasibility, acceptability, and effectiveness of iGeriCare, a web- and email-based dementia care partner educational intervention.

### Specific Objectives

The specific objectives of this study are to evaluate the feasibility and care partner acceptance of the intervention and other study methods and to examine the effectiveness of the educational resources on family care partners’ knowledge, self-efficacy, and sense of burden.

### Research Questions

This study aims to answer the following research questions: (1) Is the intervention feasible for participants? (2) Are participants engaged and satisfied with iGeriCare? (3) Does iGeriCare increase knowledge of dementia? (4) Does iGeriCare increase self-efficacy? and (5) Does iGeriCare decrease burden?

## Methods

### Study Design

This study is a 2-arm, pilot, feasibility RCT with intervention and control groups. Participants will receive assessments at baseline (T1) and 8 weeks (T2).

### Research Platform, Learning Management System, and Email Campaign

Developers in the Division of e-Learning Innovation at McMaster University created a web-based research platform capable of hosting participant eligibility screening, informed consent, and block randomization. Microlearning email campaigns were set up in the MailChimp email marketing and automation platform, with a campaign to schedule 2 emails per week to each participant to reinforce the learnings from either the intervention or control e-learning. A total of 2 courses (intervention and control) were set up in the Litmos learning management system to host the prestudy surveys, e-learning lessons, and poststudy surveys for each group.

### Participants

#### Inclusion and Exclusion Criteria

Individuals who meet the following self-reported inclusion criteria will be invited to participate in the study: (1) family or friend care partner of a person living with dementia, (2) reside in Canada, (3) 16+ years of age, (4) able to read and listen to English, (5) access to email and internet, and (6) comfortable using email and internet. Participants who answer “yes” to all 6 questions using the research platform will be invited to read the study information and provide informed consent before creating a participant account.

#### Sample Size

A total of 125 participants will be recruited in anticipation of a dropout rate of up to 30%, based on retention rates from previous trials on web-based interventions for our target population [10,25-27]. We expect a final sample size of 80 (n=40 per group), which will be sufficient to assess the feasibility and acceptability of the intervention.

#### Recruitment

Facebook digital marketing advertisements will be used to recruit potential participants on a rolling basis until 125 eligible participants provide informed consent. Facebook has been found to be an increasingly effective and low-cost tool to drive the digital recruitment of research study participants [28]. Using targeted audience demographic selection, only persons who have been predetermined by Facebook to have “interest” in relevant topics (ie, Alzheimer disease, dementia, and caregiving) will receive study advertisements.

#### Randomization and Intervention Assignment

Following account creation, the research platform will automatically randomly assign participants either to the intervention group or the control group using stratified permuted block randomization with variable block sizes of 4, 6, and 8 (randomly arranged) in a ratio of 1:1, based on care partner type (care partner to a spouse, care partner to a parent, and care partner to someone other than a spouse or parent), and level of education (high school equivalent or less, some college or university, and college or university or graduate degree).

Once a participant is randomly assigned to either the intervention or control group, an application programming interface (API) will be used to assign each participant to the matching microlearning email campaign and course in the learning management system. Participants will receive an account verification email and once verified can begin to access their assigned surveys and web-based course (prestudy surveys, e-learning lesson[s], and poststudy surveys).

#### Participant Support

Participants will be invited to email the research team at any time throughout the study period. A member of the research team will respond to all inquiries within one business day.

### Intervention and Control Groups

#### Baseline Assessments (Prestudy Surveys)

Both the intervention and control groups will receive access to the same prestudy surveys at T1 (baseline). The prestudy surveys will be used for the baseline assessments of participant demographics, knowledge, perceived self-efficacy, and perceived burden. Prestudy surveys will be identical for both the intervention and control groups.

#### e-Learning Content

**Figure 1:**
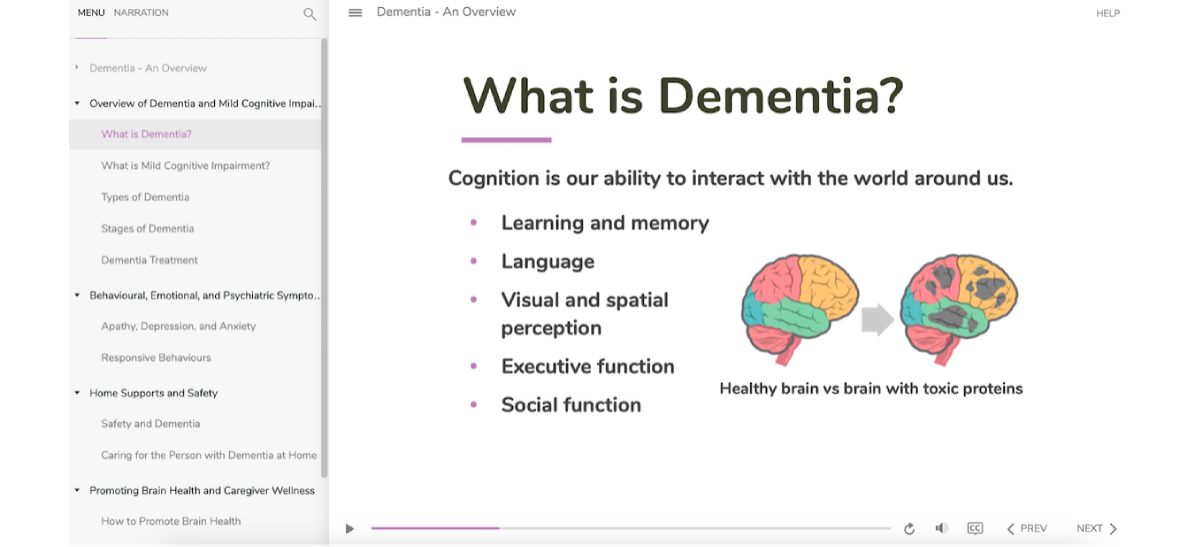
e-Learning content of the intervention group.

After completion of the prestudy surveys, the intervention group will receive access to 10 selected e-learning lessons from iGeriCare (Figure 1). The control group will receive access to a single e-learning lesson on “Promoting Brain Health.” Each group will be provided access to the other group’s e-learning content at the conclusion of the study. Specific lesson titles and outlines for the intervention and control groups are displayed in Table 1.

**Table 1:** Study lesson titles, descriptions, and group assignment.

Title of lesson	Duration (min)	Lesson content	Group assignment
What is dementia?	10-15	Understand what cognition is and how it is impacted by dementia. Learn about the most common causes of dementia and other possible causes of dementia-like symptoms.	Intervention
The different types of dementia	20-25	Learn about the similarities and differences between Alzheimer disease, vascular, Lewy body, frontotemporal, and Parkinson disease dementias.	Intervention
Stages of dementia	20-25	Explore the 3 stages of dementia. Understand how the disease progresses and know what to expect.	Intervention
How is dementia treated?	15-20	Look into the difference between managing the symptoms of dementia and modifying the disease itself. Investigate what prescription drugs and other therapies are available.	Intervention
Safety in dementia	15-20	Become aware of the risks of wandering, driving, fire, improper use of medications, and managing personal finances. Discover strategies to reduce the possibility of harm.	Intervention
Caring for the person with dementia at home	20-25	Learn about the types of services available in your home and your community from both publicly funded sources and private companies.	Intervention
Apathy, depression, and anxiety in dementia	25-30	Learn how these psychiatric issues may affect people with dementia. Understand how to cope and discover what treatments may be available.	Intervention
Behavioral, emotional, and psychiatric symptoms in dementia	35-40	Identify the behavioral, emotional, and psychiatric signs and symptoms associated with dementia. Discover strategies and resources to help to manage responsive behaviors.	Intervention
Caregiver wellness	10-15	Understand why caregiver wellness is so important. Learn what you can do to help yourself and find support.	Intervention
Dementia: an overview	10-12	Get a quick overview of dementia in this summary that covers the highlights of all our lessons.	Intervention
Promoting brain health	25-30	Explore how diet, exercise, blood vessel health, lifestyle choices, brain and social activity, and other health conditions affect brain health.	Control

### Microlearning Email Campaign

Once randomly assigned, participants will receive their designated microlearning campaign. The microlearning campaign is intended to reinforce learnings from the primary e-learning content and is customized to each group. Emails will be automated to be sent twice weekly.

### Poststudy Surveys

Poststudy surveys will be released to each participant at T2 (8 weeks) and remain open for 2 weeks. Poststudy surveys will not be released to participants who did not complete the prestudy surveys. Participants in the intervention group will be invited to participate in a follow-up qualitative interview with the research team.

### Outcome Measures

Our primary outcome is to evaluate the feasibility of implementation, including study methods, of the iGeriCare e-learning content within a clinical trial context. The secondary outcome is to determine the effectiveness of the intervention on knowledge, self-efficacy, and care partner burden.

### Feasibility of Implementation of the Intervention

We will examine intervention adherence as measured by lesson completion data and email open rates, level of technical and participant support required from the research team including tracking and sending reminders, participant satisfaction survey ratings, and exit interview data from a sample of participants from the intervention group.

### Feasibility of the Study Methods

Study methods will also be evaluated. We will examine recruitment rate as measured by participants per day, participant acceptability of the intervention and surveys as measured by completion and attrition rates, time to complete the intervention and surveys, level of engagement and exposure to the intervention (including potential “fast trackers” moving through the intervention at a high speed), survey data, and exit interview data.

### Clinical Effectiveness Outcomes

#### Knowledge

Knowledge will be measured through the Dementia Knowledge Assessment Scale (DKAS; reliability α=.85; ωh=.87; overall scale) [29]. The DKAS consists of 25 items on different aspects of dementia that could be answered with “True,” “Probably True,” “False,” “Probably False,” or “I don’t know.”

#### Self-Efficacy

Self-efficacy will be measured through the Revised Scale for Caregiving Self-Efficacy (RSCSE; reliability α≥.80) [30]. The RSCSE contains 15 items within 3 subscales (self-efficacy for obtaining respite, responding to disruptive patient behaviors, and controlling upsetting thoughts about caregiving).

#### Burden

Burden will be measured through the Zarit Burden Interview (ZBI; reliability α=.92) [31]. The ZBI contains 22 items. Each item on the interview is a statement that the caregiver is asked to endorse using a 5-point Likert scale. Response options range from 0 (“Never”) to 4 (“Nearly Always”).

### Qualitative Interviews

A sample of 10 consenting participants from the intervention arm will also be invited to participate in a 10-minute semistructured telephone interview looking for their impressions about their experience, user acceptance, barriers, and facilitators related to the educational tools and the intervention. This will allow the participants to have the freedom to express their experience, as opposed to having closed-ended questions. While we anticipate interviewing approximately 10 participants from the intervention group, we will also look for saturation of themes or feedback to determine when to terminate interviews. Interview items will be based on a participatory knowledge translation framework and mixed methods informed by the Consolidated Framework for Implementation Research (CFIR) to evaluate factors that could influence implementation effectiveness. The qualitative interview guide can be viewed in Multimedia Appendix 1.

### Data Analysis

Blinded data analysis will be performed upon data collection for both cohorts. The CONSORT (Consolidated Standards of Reporting Trials) extension for randomized pilot and feasibility trials as well as CONSORT-EHEALTH will be used for final reporting [32-34].

### Participant Characteristics

Differences in participant demographics between groups will be assessed using unpaired 2-tailed *t* tests. Both stratification factors, care partner type (care partner to a spouse, care partner to a parent, and care partner to someone other than a spouse or parent) and level of education (high school equivalent or less, some college or university, and college or university or graduate degree), will be included in all models. In addition, age, sex, education, stage of dementia, and length of time as a care partner will be looked at as possible effect modifiers of the relationship between the group and the outcomes, and timepoint and the outcomes.

### Feasibility of Implementation and Study Methods

Quantitative feasibility-related outcomes (eg, email open rates and participant satisfaction survey ratings) will be compared between groups using unpaired 2-tailed *t* tests. Qualitative feedback from postcourse surveys and semistructured interviews will be coded, themed, and analyzed using a generic qualitative approach [35]. Qualitative content analysis will focus on the identification of barriers and facilitators to the adoption of iGeriCare e-learning, participant perceptions of the intervention, whether they felt it met their needs, and other experiences with the intervention, outcome measures, and study protocol.

### Knowledge, Self-Efficacy, and Burden

Linear mixed models will be used to assess the relationship between group assignment and score for each of the outcomes: the DKAS and its subdomains (causes and characteristics, communication and behavior, care considerations, and risks and health promotion), the RSCSE and its subdomains (obtaining respite, managing disruptive behaviors, and controlling upsetting thoughts), and the ZBI. All models will include the 2 stratification factors, education level (high school equivalent or less, some college or university, and college or university or graduate degree) and care partner type (care partner to a spouse, care partner to a parent, and care partner to someone other than a spouse or parent). Group assignment will be included as the primary independent variable of interest with interaction from time to determine how the relationship between outcome score and group assignment will change from baseline to follow-up. Potential effect modifiers, including age (categorized as <44 years, 45-64, and ≥65 years), gender, education level, stage of dementia (mild [early-stage] dementia, moderate [middle-stage] dementia, severe [late-stage] dementia, or “I don’t know”), and length of time as a care partner (less than 3 months, more than 3 months but less than 1 year, more than 1 year but less than 2 years, more than 2 years but less than 5 years, or more than 5 years), will be explored in relation to group assignment. These variables will be retained in the model if the likelihood ratio test indicates a significant difference between the nested model and the full model (*P*<.05). If they are not significant modifiers of the group-outcome relationship, then they will be investigated as effect modifiers with respect to time. An intention-to-treat analysis will also be performed and include all individuals as per group assignment. A per-protocol analysis will also be performed. Correlations between the number of lessons in the intervention completed and the DKAS score will be analyzed.

We will also look for inverse correlations between the DKAS and ZBI scores, as well as between the RSCSE and ZBI scores. Analyses will be performed using an α level of .05 and 2-sided tests. All analyses will be conducted using RStudio software (Posit).

### Qualitative Interviews

A conventional inductive content analysis approach will be used [36]. Data will be systematically examined. Codes and themes will be generated based on the content, with no reliance on preconceived theories. Weekly meetings will be held with the research team and analyst to examine themes, validate the coding process, and maintain consistency and depth in data interpretation.

### Ethical Considerations

This study gained Hamilton Integrated Research Ethics Board approval on November 21, 2022 (project #14096) and was registered on ClinicalTrials.gov (identifier NCT05114187). Participants were required to provide informed consent and were informed of the length of time of the e-learning, surveys, and email campaign; they were also informed about the details surrounding data collection, storage, and investigator identities. Participants’ identities and confidentiality were maintained throughout the research study. All participant data were deidentified and all findings will be nonidentifiable. There is no known risk or harm to participating in this study or publicizing its results or findings. Participants in run 1 were provided with a CAD $40 (US $28.6) Amazon gift card, and participants in run 2 were provided with AskingCanadians points that can be redeemed for various gift cards as compensation for participation in the study.

## Results

### Initial Cohort Participants

**Figure 2:**
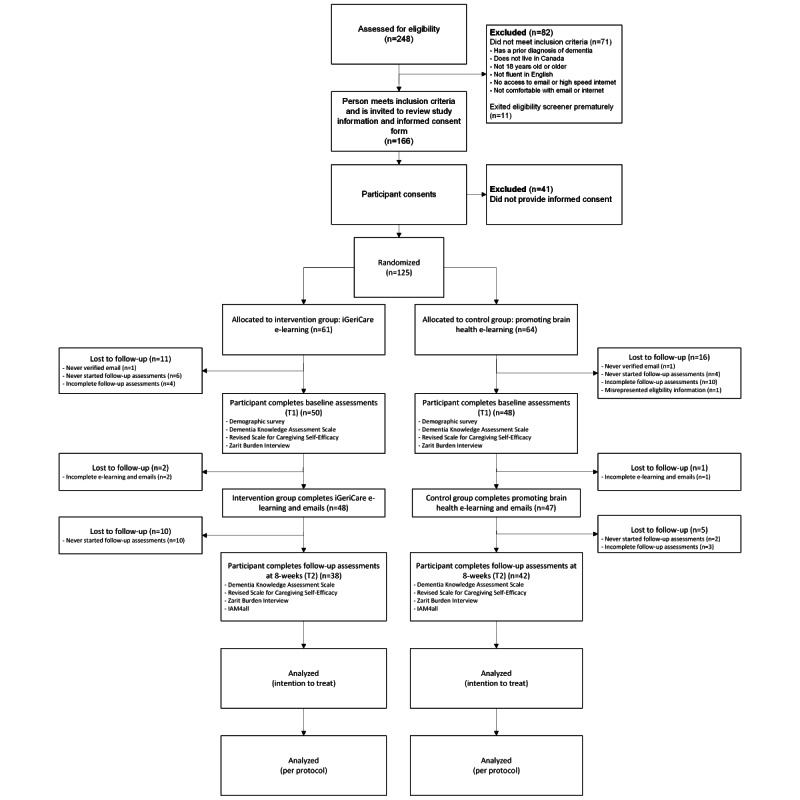
Study flow diagram for run 1.

Recruitment began on September 6, 2022, and finished on October 2, 2022. A total of 125 participants were randomly assigned to the intervention (n=61) or control (n=64) group, and data collection concluded in January 2023 (Figure 2).

Preliminary results showed a substantial number of participants who did not engage with the protocol as initially intended. A number of participants were identified as not having completed the protocol as intended, as demonstrated by “fast-tracking” through the e-learning (ie, clicking next without allowing time for reading or listening to content) or by repeatedly clicking “I don’t know” on prestudy or poststudy surveys.

Following this, a decision was made to recruit an additional cohort of participants (n=100) using learnings gained from the initial recruitment cohort concerns. A paid panel service, AskingCanadians, was engaged to assist with recruitment. AskingCanadians is a reputable paid panel service that was able to specifically target family and friend care partners of people living with dementia.

### Second Cohort Participants

**Figure 3:**
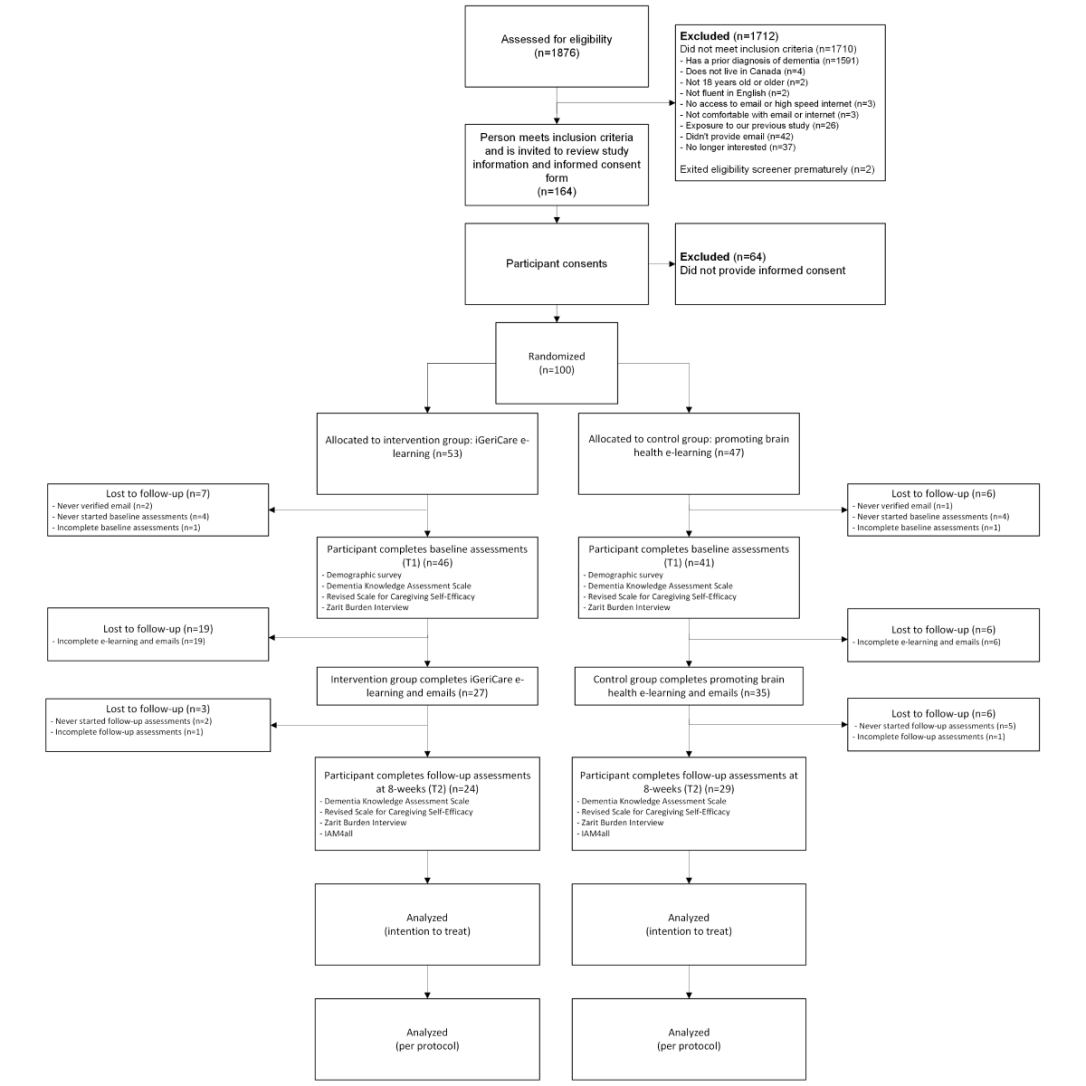
Study flow diagram for run 2.

Recruitment for the second cohort began on June 12, 2023, and concluded on June 27, 2023. A total of 100 participants were randomly assigned to either the intervention (n=53) or control group (n=47) group; data collection concluded in September 2023 (Figure 3).

### Future Results

Results from run 2 of the RCT will be analyzed in spring 2025, published in peer-reviewed journals in the field of aging and geriatrics, and presented at relevant conferences in fall 2025. Results will report on the overall feasibility of implementation of iGeriCare e-learning components in a clinical trial context; care partner acceptance of the intervention (including study methods and participant process); as well as any changes in knowledge, self-efficacy, and burden.

## Discussion

### Overview

The primary objective of this pilot study is to evaluate the feasibility of implementing the iGeriCare e-learning content within a clinical trial context; the secondary objectives include evaluating the feasibility of the study methods and the effectiveness of the intervention. The results of this pilot feasibility study will contribute to the planning of a larger RCT in the future. The iGeriCare RCT has several strengths. First, because the study intervention is freely available on the web, it would be easy to implement, scale, and spread as part of a broader knowledge translation strategy. Second, the intervention is also available in both English and French, which is important for broader dissemination nationally within Canada. Third, while web-based dementia care partner interventions have been previously shown to be effective, those that incorporate evidence-based principles of multimedia instructional design are likely to prove even more effective.

### Limitations

There are some limitations to this work. First, while Meta advertising proved to be an extremely effective and timely recruitment strategy, we did not anticipate the volume of noncompliant (“fast tracker”) and “fraudster” participants that would be recruited during the initial recruitment phase [37]. It is possible that the participation incentive caused some users to declare that they are dementia care partner in the eligibility screener in order to register for the study. To remedy this, a second cohort of participants was recruited through the use of a reputable paid panel service, AskingCanadians. Future studies will look at redesigning a social media recruitment campaign to discourage noncompliance or fraudulent participants. Second, this trial does not look at the long-term impact of web-based education and potential changes to knowledge, self-efficacy, and burden. Future work could consider incorporating additional time points to assess longer-term impacts. Finally, although AskingCanadians was enabled for recruitment of the second cohort of participants, there is still the potential for selection bias and exclusion of those who are lower income or do not have regular access to technological resources. Future studies may explore the barriers to access and look at providing other avenues to web-based resources for disadvantaged populations.

### Conclusions and Next Steps

The expected outcomes related to feasibility are participant acceptance and engagement with the intervention and the study protocol. We anticipate a trend toward an increase in knowledge and self-efficacy, and a reduction in burden in the intervention group, although the study was not powered to fully assess effectiveness. We expect that the study will have implications for future e-learning research with care partners of people living with dementia.

The results of this study will contribute to the planning of a larger RCT in the future, as well as the evaluation of innovative, cost-effective, and efficient dementia care partner resources that can complement traditional approaches. A final manuscript will be prepared to share all results in fall 2025.

## Appendix

Multimedia Appendix 1 Qualitative interview guide.

Multimedia Appendix 2 Peer review report from the PSI Foundation (Ontario, Canada).

## Abbreviations

API: application programming interface
CFIR: Consolidated Framework for Implementation Research
CONSORT: Consolidated Standards of Reporting Trials
DKAS: Dementia Knowledge Assessment Scale
RCT: randomized controlled trial
RSCSE: Revised Scale for Caregiving Self-Efficacy
ZBI: Zarit Burden Interview

## Data Availability

The datasets analyzed during this study will be available from the corresponding author on reasonable request once data collection is complete.
