# Supplementary material for: Web-Based Education Program for Care Partners of People Living With Dementia (iGeriCare): Protocol for a Pilot Randomized Controlled Trial
Source: JMIR Res Protoc. 2025 Jun 4;14:e67048. doi: 10.2196/67048 (PMC12177419; doi:10.2196/67048)
Supplement: Multimedia Appendix 1 [file resprot_v14i1e67048_app1.docx]

**Semi-Structured Interview Guide** - to be used as a template when performing interviews

Interview guide is based on the CFIR Guide for conducting interviews.

**Inner Setting**

Intervention Source

1. Who developed the intervention?
   1. What is your opinion about the e-learning lessons and emails that were developed by experts from McMaster University?
   2. Did the fact that it was developed by experts from McMaster University encourage you to sign up for the study and/or engage with the e-learning? How did the Amazon gift card incentive affect your decision to sign up and continue the study?

Relative Advantage

1. Have you completed other e-learning about dementia?
   1. How does this e-learning compare to other ways you might have learned about dementia previously, such as other e-learning, websites, or talking with a doctor?
      1. Advantages
      2. Disadvantages

Adaptability

1. Would you make any changes to the e-learning?
2. Would you make any changes to the study as a whole, from sign-up to the final surveys?

Complexity

1. Did you find the e-learning complicated to navigate?
2. Did you find the study itself complicated to navigate, such as signing into the research platform and determining your next steps?

Design Quality & Packaging

1. What did you think of the design of the e-learning and the emails you received in terms of their format, such as their visual appeal and the length of the lessons?
2. Did you find you needed any other help or support to use the e-learning lessons?

**Outer Setting**

Patient Needs & Resources

1. Did the e-learning meet your needs?
2. Do you think the e-learning would meet the needs of other care partners?

**Characteristics of Individuals**

Knowledge & Beliefs about the Intervention

1. What’s your opinion, in general, about the use of online learning for care partners of people living with dementia?
   1. Would you structure the e-learning differently, or is there a different format you might prefer altogether?

Self Efficacy

1. Did you feel confident using this e-learning?
2. Do you plan to continue using this e-learning?

Individual Stage of Change

1. Would you refer others to this e-learning?

**Process**

Planning

1. How did you complete the e-learning (i.e., did you schedule time to complete)?

Engaging

1. Who would be important for you to tell about this e-learning? (i.e., your doctors, your family, your friends)
2. How would you let others know about this e-learning?

Executing

1. Were you able to complete the e-learning?
   1. If yes, what helped you to stay on track to complete it?
   2. If no, what were some of the barriers that prevented you from completing?
2. Were you able to check out the emails you received twice per week?
   1. What did you think of their frequency?
3. What did you think of the surveys you completed at the beginning and end of the study?
   1. Do you feel they took too little time, too much time, or just about right?
