# Supplementary material for: Web-Based Education Program for Care Partners of People Living With Dementia (iGeriCare): Protocol for a Pilot Randomized Controlled Trial
Source: JMIR Res Protoc. 2025 Jun 4;14:e67048. doi: 10.2196/67048 (PMC12177419; doi:10.2196/67048)
Supplement: Multimedia Appendix 2 [file resprot_v14i1e67048_app2.pdf]

Reviewer Number: OR1

Resubmission of Previous Application: Yes

Summary: Several new members have been added to the team including Dr. Markle-Reid who has significant experience in the implementation of complex patient-centred care interventions to improve outcomes of older adults and their care partners - she holds a Tier 2 Canada Research Chair. There are also several geriatricians and family physicians, a fellow in geriatric psychiatry, a neurologist and a biostatistician to complement the expertise and experience of Dr. Levinson and Dr. Sztramko.

Evaluation: The addition of the Co-Is as noted above rounds out the collective qualifications, experience and past experience to successfully carry out this project.

Originality: The originality has been improved with a greater focus on iGeriCare as a caregiver-based intervention. Importantly, caregivers were involved in the development process including the design and validation of lesson topics.

Feasibility: The recruitment period has been lengthened so that the target is enrolling 2 participants/week. The staffing for enrolment and carrying out the different tasks in the study protocol reflects the experience of this study team for a successful outcome of the project. Feasibility will be further evaluated in this pilot project and changes to the study protocol made in preparation for a randomized clinical trial.

Clinical Relevance: Family caregivers of persons living with dementia have significant needs in terms of timely access to information as care needs change with progression of dementia. Timely access to this information is critical and not something that can wait for the next visit to a health care professional. Further, this intervention offers more of a public health approach to timely access to information about dementia. The cost:benefit of this approach to informing care partners is likely to be very high and would reduce pressures on health care professionals to provide timely access to one-on-one interactions to address the caregiver's needs.

Research Design and Methodology: The design has been modified from a pre/post analysis to a randomized pilot study comparing iGeriCare to another validated online dementia care resource. The intervention will be evaluated according to its feasibility and care partner acceptance and it impact on care partner self-efficacy, knowledge and burden of care. Post-study interviews will provide an opportunity for care partners to reflect on their experience with the educational program.

Likelihood of project being completed in time stated: With the addition of Co-Is and modifications made in this revised protocol, there is a high likelihood of timely completion of the project.

Appropriateness of the literature review: A comprehensive review of the literature is appropriate for this

proposal, particularly highlighting the limited focus on the role of caregivers in previous publications and the cost:benefit and ease of access to and utility if the iGeriCare online platform.

Budget: Appropriate for the proposed research - no changes to suggest.

Overall Comments (if applicable, appropriateness of this project for resident research): This is an excellent proposal for a pilot study that will lay the groundwork for a randomized trial of the iGeriCare intervention. The CFIR framework is a state-of-the-art implementation method and will support the successful completion of the objectives for this project.

Reviewer Number: OR2

Resubmission of Previous Application: Yes

Summary: The applicants have gone to great lengths to address previous comments of the original submission. The application is well-written and well-supported with the research literature.

Evaluation: The research team is made up of accomplished physicians with extensive experience with medical student e-learning, technology-based interventions, as well as works with older adults and various chronic conditions. Respectfully, the missing link for me is with respect to the lack of (continued) collaboration of family carers or including the support of the Alzheimer Society of Canada (and/or Ontario) in the current project.

Originality: The applicants have done a good job of explaining the uniqueness and potential benefit of an intervention like iGeriCare. A web-based, individual intervention has potential to support some family carers and the design of the current application may identify who iGeriCare helps and for whom the intervention is not useful.
It is important that at the end of the project the control group will also have access to the iGeriCare, a wonderful consideration (and one with ethical implications).

Feasibility: The applicants have addressed several concerns expressed in their previous submission. The addition of several new physicians (including geriatricians) has been helpful and Dr. Markle-Reid is a strong nurse researcher. These new (7) members can support the conduct of this pilot project. However, a family carer or collaboration with the Alzheimer Society was not included – a missed opportunity. Kindly note, the Alzheimer Society of Canada has a research portal that can assist projects like iGeriCare to recruit potential participants and advertise the study widely among those who would benefit from iGeriCare.

Clinical Relevance: The aims of this current application will support the outcome of clinical significance.

It will be important to hear/understand if iGeriCare is a suitable educational resource in contrast to in-person, clinician-led education. COVID-19 has certainly revealed the need for and importance of web-based interventions for all kinds of caring contexts. A key consideration is the limit of having ready access to a computer and the internet – not an option for all people living in the context of dementia. Some discussion of why the study groups will be stratified by level of education is warranted. I note there is a great deal of medical terminology in the figures that depict the iGeriCare lessons; such reliance on this heavy terminology may limit who will gain an understanding of dementia from the proposed intervention.

Research Design and Methodology: The applicants have gone to lengths to increase the rigour of their approach and as such readily justify the funds they are requesting. This re-submission is meant to support the conduct of a larger RCT design in future. Most importantly, the current application focuses on data collection from family carers alone, which is helpful to hear directly from the people who will benefit from iGeriCare. I wonder if 3 months from baseline might be a better timeframe to use the intervention. Having 2 months to complete 10 sessions and bi-weekly (email) micro-sessions might be a lot to ask of some family carers.
Inclusion of a qualitative semi-structured interview with a sample of participants is a strength of this currently proposed approach. Respectfully, the suggestion of 10 minutes for this interview is likely not feasible; please consider at least 30 minutes. A sample of the questions to be asked would have been helpful.

Likelihood of project being completed in time stated: The revised timeline for this re-submission is feasible and well thought-out. The addition of another 12 months was a wise consideration

Appropriateness of the literature review: The applicants have extended the literature review beyond the original application. Incorporation of the work by Levinson and colleagues (2020) supports the proposed work. Much of the information regarding people living with dementia and their family carers, however, is quantitatively-based to describe the context and is lacking in the (qualitative) perspective of the family carers. It would be helpful to have a more central focus on the needs and concerns of family carers and how iGeriCare would benefit them (at the moment the time and effort of health care providers seem to be the focus).

Budget: Justification of budget allocation is reasonable and thorough. Detail around the duties and roles of the RA and coordinator are well done.

Overall Comments (if applicable, appropriateness of this project for resident research): Thank you for the opportunity to review this resubmission.

Reviewer Number: 3

Resubmission of Previous Application: No

Summary: Online resources to improve care partner education for families living with dementia is a key need and these resources need to be evidence-based and tested. This is a grant application to fund a 3-year, two-arm Pilot Randomized Controlled Trial of an (already existing) Internet-Based Education Program for Care Partners of People Living with Dementia (iGeriCare). RCT outcomes are 1) the feasibility and care partner acceptance of the intervention and key components of the study methods (participant recruitment strategies, retention, acceptability of the intervention and outcome assessment surveys), and 2) the impact of iGeriCare on care partner self-efficacy, knowledge, and sense of burden.

Evaluation: Led by a team of clinician researchers who are, in my view, well qualified and the right people to lead this project. PI Levinson is a psychiatrist and Director, Division of e-Learning Innovation and John R. Evans Chair in Health Sciences Educational Research
Co-PI Sztramko is a geriatrician academic. Both have been involved and published in this field as lead authors. The co-investigator team is a well-built team for this project, with representation from established and 2 early career investigators on the team.
The research team of (all part-time) RA, research coordinator, educational technologist, digital marketing specialist and lead web developer is a lean team, but I assume the PI and co-investigator team will provide in-kind time.
The intervention (internet resource iGeriCare, has already been developed and has been in use. For future studies, consideration of a wider national and even international team could be considered for further testing and roll-out.

Originality: Whilst there are several family carer interventions, there is a dearth of interventions that are co-designed with the population that it is meant to target. IGericare, whilst not entirely co-designed, at least involved caregivers in the development process, including in the design and validation of the lesson topic selection, as well as iterative review of each and every lesson. For this reason, iGeriCare is original and needs an evidence-base, which this grant would provide via a pilot RCT. iGeriCare also has other points of originality and difference which are important (asynchronous delivery, accompanying email-based micro-learning) to formally evaluate.

Feasibility: The team aim to recruit 125 participants and are anticipating a dropout rate of up to 30%, with a goal of a final sample size of n=80 (n=40 per group). They aim to recruit approximately 2 caregivers per week over a 78-week rolling recruitment period. In my opinion, this is feasible in the context of this three year project and the identified enrollment period, online intervention, and Investigator networks.
Timelines: If the timelines for Phase 1 and/or Phase 2 blow out, then the Phase 3 time (a fairly generous -12 months) can be used.

Clinical Relevance: This intervention is already being "prescribed" in clinical practice in Canada, but there have been no clinical trials to show effectiveness. If this RCT found iGeriCare to be feasible, acceptable, and improve care partner self-efficacy, knowledge, and sense of burden, then the intervention would have

an evidence-base, and also inform the design of a future, larger RCT. Providing evidence-based internet-available education that has been shown to help care partners improve knowledge, self-efficacy, and well-being will be of great clinical relevance (and also economic relevance).

Research Design and Methodology: This study is a two-arm pilot randomized controlled trial. Participants will receive assessments at the following timepoints: baseline (T1) and at 2-months (T2). This is robust enough for a pilot RCT, and the selected outcome measures are appropriate. I do have concerns about the following:
1. Are you measuring the dementia stage of the care givers care recipient? The stated outcome measures are likely to be affected by staging, severity, and types of signs and symptoms, and diagnosis type.
2. Reach to rural/ isolated participants, and those from more diverse backgrounds?
3. Would recommend the investigators also collect "length of caring role" and consider stratification based on this
4. Consider adding a follow up time point to look at stability of effect
5. Consider subgroup analyses of the primary outcome by geographic and socioeconomic classification.
6. The gathering of qualitative data within 10 minutes seems rushed, for any population. Looks also like a content analysis is warranted rather than a grounded theory approach as stated (I don't think the investigators are intending to discover or construct theory?).

Likelihood of project being completed in time stated: Recruitment targets seem reasonable in the time period listed, but a risk management plan in the event of poor recruitment would be appreciated (this is a difficult cohort to engage in research). The intervention is already developed. The team has worked together before. A few concerns about budget and research team EFT, as noted in the below section on budget.
Timelines: If the timelines for Phase 1 and/or Phase 2 blow out, then the Phase 3 time (a fairly generous -12 months) can be used.
Overall, no particular concerns about project being completed within 3 years.

Appropriateness of the literature review: The literature review was appropriate, but more detail wold have been appreciated and would have strengthened the proposal. For example, it did not review what is already available and/or trialed in recent years (e.g. iSupport, START, Dementia Companion), and what the gaps are in the existing evidence-base and intervention library. The literature review also did not focus on evidence for multi-component interventions, nor did it justify the use of single person vs group format for iGeriCare.

Budget: The research team of (all part-time) RA, research coordinator, educational technologist, digital marketing specialist and lead web developer is a lean team, but I assume the PIs and co-investigator team will provide in-kind time. The budget of GRANT TOTAL: $240,364.94 presents value for money for such a project, and leverages an already-developed intervention and team.

Overall Comments (if applicable, appropriateness of this project for resident research): Overall, a well developed project with pilot data and publications, with an appropriate methodology for a pilot RCT

evaluating an already-developed intervention that is already being used and prescribed clinically. The PIs and Co-investigator team are the right people to lead this Canadian project, which will likely lead to further research in the important area of carer interventions, and further testing and validation of the iGeriCare intervention. If found to be effective, this will have impact on the health and well-being of care givers of people living with dementia, with clinical relevance and perhaps economic relevance.

Reviewer Number: 4

Resubmission of Previous Application: No

Summary: There has been an increase in the use of online interventions for family carers but there is a lack of high quality evidence and RCTs to evaluate their effectiveness. Due to the covd-19 pandemic many face to face services are no longer available, and online support also has the benefit of being substantially cheaper to the health care system. This project is testing the feasibility of the use of an online education programme for family carers of people living with dementia. Through a 2 armed randmoised feasibility study, they are exploring feasibility of research methods and acceptability. Participants will be randomised to the intervention arm or a control and data collected at two time points two months apart.

Evaluation: This was very difficult to ascertain! There is Limited information is provided on the team in the main application which is disappointing, based on qualifications and positions we can assume they are experienced but little detail is given on specific skills instead it is generic stating for example they will review protocol. I question experience of qual methods and also feasibility studies as some of the statements are more appropriate for a full rct not feasibility work.

Looking at the (very long!) CV's of the principal investigators have limited experience so suggest they may need a fair amount of mentoring and support but there are other members of the team that will be able to provide this.

It is very difficult to look through 134 pages of CV and try pull out overall team experience. Apologies to applicants if this was requested by PSI but this was very difficult and could have been done better to highlight experience and abilities

Originality: There is more and more work being conducted in this field, however this seems to be a good education programme and is worthy of good evaluation. The team seem passionate and have conducted previous work on this programme. There are still limited established programmes and so testing this is good value for money rahter than recreating another programme.

Feasibility: this seems feasible although suggest sample size is reduced. Interviews need to be longer

and a greater focus for data on acceptability and feasibility generally.

Clinical Relevance: This is a highly clinically relevant project. As more and more people age and numbers of people living with dementia increase, the burden on family carers will increase dramatically. This has been highlighted this year in the pandemic and online support has been invaluable to so many people. We need to future proof support and this study offers a way to do that in a meaningful way. The project is led by clinicians who are able to see first hand the clinical relevance and can ensure it remains grounded in clinical relevance.

Research Design and Methodology: The methodology seems appropriate and on the whole well thought through. 125 partcipants is large for a feasibility trial however. There is no set rule for this however a rule of thumb for feasibility is generally 50 as you are not looking to determine effectiveness but more looking at feasibility of the study methods and acceptability of the intervention.

The 'exit interviews' which is really about the intervention and a process evaluation is small for this size of study. 10 interviews should be sufficient however 10min is greatly underestimated - these are more like going to be 60min if done properly. A grounded theory is not appropriate and is not possible from 10 interviews of 10min each. I think there needs to be greater input/expertise on qualitative methods - this is a vital part of the feasibility and seems to just be bolted on with little thought or experience. Content analysis is also mentioned so i am a little conused.

All the hypotheses are not needed/appropriate for feasibility studies.

I would have encouraged a third follow up to look at the effects of the intervention beyond immediate completion - i.e. around 6 months. This would be vital in a main trial I think and therefore testing feasibility of collecting this now would be essential.

The analysis plan for measures is quite detail, which is good, however most you can do is descriptive with these numbers and again the aim of it being feasibility. I am worried the authors think they can make bigger knowledge claims than they can with this study.

Likelihood of project being completed in time stated: i think this is doable but the timelines seem odd - little time is given for phase 1a whihc i suspect will take longer, while phase 2 is given 18 months to recruit - i agree this does take a lot of time but suggest the number needed to recruit is much less than applicants plan and also the intervention is only 2 months. Also think phase 3 is long.

Appropriateness of the literature review: The literature review on the whole is well conducted. There are more interventions which could have been discussed, in particular there are interventions from the netherlands and the uk which could have been flagged giving more specific details of how this intervention differs. I note the applicants pointed out they expanded on this based on the previous review, although i think this case could be stronger.

Budget: this looks fine.

Overall Comments (if applicable, appropriateness of this project for resident research):

Reviewer Number: 6

Resubmission of Previous Application: No

Summary: Carers of people with dementia are often exposed to significant and sustained mental and physical stresses and can therefore become disempowered, stressed and face high high levels of anxiety and depression. This project aims to undertake a pilot randomised control trial of iGeriCare for informal (unpaid) caregivers of people with dementia. 125 participants will be randomised either to receive a "promoting Brain Health" eLearning programme or the iGeriCare platform. The project will assess three key outcomes: self-efficacy, as measured by the Revised Scale for Caregiving Self-Efficacy (RSCSE); knowledge, as measured by the Dementia Knowledge Assessment Scale (DKAS); and decrease burden, as measured by the Zarit Burden Interview (ZBI). Participants will be exposed to the platform for a total of eight weeks

Evaluation: The expanded team now includes much more dementia experience compared to previous submissions. I have no concerns about their ability to conduct the work suggested although it might be important to have dedicated members of staff who are statisticians to support the aim of a larger future study.

Originality: I understand that from the previous review stage originality has been previously raised as a discussion point. Further context on this could be very useful- there are a number of innovations going on around the world just now, not least those developed at the World Health Organization (iSupport, https://www.who.int/publications-detail-redirect/9789241515863) including cultural adaptation in various languages.

Further it would be interesting to clearly state within the application the scientific rationale, and specific origin of the module content so a true comparison can be made with other existing interventions.

Feasibility: In my experience one of the greatest challenges in dementia/carer studies is that of recruitment. Given that the authors state that the authors have had over 140,000 unique visitors to the IGeriCare, I don't see any issues on recruiting 2 caregivers per week into this study.

Clinical Relevance: The study follows outcomes that are extremely relevant to the dementia caregiver population. However, it is not clear to me why the authors target these. More information on the design of the intervention background theory would help justify the clinical relevance.

Research Design and Methodology: Overall, the improve randomised control trial pilot is a real strength of the work. I would suggest however a number of small improvements:
- good command of the english language could become quite vague as an inclusion criteria- more specific would be to exclude individuals unable to understand written english
- Excluding those who are not comfortable using email and internet may question the external validity of the pilot (and main study)- is it not possible to provide limited technical support within the research team or with technical colleagues?
- Given the high usage of the platform- the authors may wish to consider making previous use of the intervention as an exclusion category?
- Some more specificity on occasion would be useful- e.g. We anticipate "high rates" of lesson completion and email opens- what would the authors deem success to look like?
- "Access to email and internet" could look quite different for your user group- does this mean you would include individuals who do not have internet in the home but are using it elsewhere? Is there a cut off for how regularly they use it?

Likelihood of project being completed in time stated: This appears likely to me- the platform is up and running, the group are really well connected to their target population.

Appropriateness of the literature review: The literature review provides a strong grasp of related works, however as mentioned above it would be useful to add more context around the background theory/behavioural change/psychology underpinnings of the work alongside and be more exhaustive in terms of similar efforts elsewhere

Budget: I think the work is very good value for money overall- the token payment for participants is a useful addition.

Overall Comments (if applicable, appropriateness of this project for resident research): Overall, I think this is much needed research. The authors have clearly put a lot of thought and planning into the application and have a clear plan for follow on implementation.
